# Advancement and Challenges in Monitoring of CAR-T Cell Therapy: A Comprehensive Review of Parameters and Markers in Hematological Malignancies

**DOI:** 10.3390/cancers16193339

**Published:** 2024-09-29

**Authors:** Weronika Ploch, Karol Sadowski, Wioletta Olejarz, Grzegorz W. Basak

**Affiliations:** 1 Department of Biochemistry and Pharmacogenomics, Faculty of Pharmacy, Medical University of Warsaw, 02-097 Warsaw, Poland; s082653@student.wum.edu.pl (W.P.); karol.sadowski@wum.edu.pl (K.S.); 2 Department of Hematology, Transplantation and Internal Medicine, Medical University of Warsaw, 02-097 Warsaw, Poland; grzegorz.basak@wum.edu.pl; 3 Centre for Preclinical Research, Medical University of Warsaw, 02-097 Warsaw, Poland

**Keywords:** chimeric antigen receptor T-cell (CAR-T), immunotherapy, monitoring, biomarkers, flow cytometry (FC), polymerase chain reaction (PCR)

## Abstract

**Simple Summary:**

Chimeric antigen receptor T-cell (CAR-T) therapy has revolutionized the treatment of hematological malignancies but is often associated with significant adverse events. As these events affect up to 80% of patients, they constitute a crucial problem to overcome. This review focuses on the monitoring process of CAR-T cell therapy including the monitoring of the persistence, activity, and phenotyping of the cells. The implementation of tools like flow cytometry and polymerase chain reaction provides insights into cellular responses, enabling the optimization of CAR-T cell therapy for more precise and personalized treatment and addressing the challenge of tumor relapse.

**Abstract:**

Chimeric antigen receptor T-cell (CAR-T) therapy has revolutionized the treatment for relapsed/refractory B-cell lymphomas. Despite its success, this therapy is accompanied by a significant frequency of adverse events, including cytokine release syndrome (CRS), immune-effector-cell-associated neurotoxicity syndrome (ICANS), or cytopenias, reaching even up to 80% of patients following CAR-T cell therapy. CRS results from the uncontrolled overproduction of proinflammatory cytokines, which leads to symptoms such as fever, headache, hypoxia, or neurological complications. CAR-T cell detection is possible by the use of flow cytometry (FC) or quantitative polymerase chain reaction (qPCR) assays, the two primary techniques used for CAR-T evaluation in peripheral blood, bone marrow (BM), and cerebrospinal fluid (CSF). State-of-the-art imaging technologies play a crucial role in monitoring the distribution and persistence of CAR-T cells in clinical trials. Still, they can also be extended with the use of FC and digital PCR (dPCR). Monitoring the changes in cell populations during disease progression and treatment gives an important insight into how the response to CAR-T cell therapy develops on a cellular level. It can help improve the therapeutic design and optimize CAR-T cell therapy to make it more precise and personalized, which is crucial to overcoming the problem of tumor relapse.

## 1. Introduction

Chimeric antigen receptor T-cell (CAR-T) therapy approved for relapsed/refractory B-cell lymphomas and acute lymphoblastic leukemia (ALL) has led to improved disease outcomes with notable success [1,2]. However, several challenges hinder the full effectiveness of CAR-T therapy, including antigen escape, limited anti-tumor activity, poor trafficking, and restricted tumor infiltration [3]. Furthermore, CAR-T cell therapy often results in adverse effects like cytokine release syndrome (CRS), immune-effector-cell-associated neurotoxicity syndrome (ICANS), and cytopenias [4]. One of the major obstacles to widespread clinical use is cancer relapse, driven by the tumor cells’ inherent factors and superior adaptability [5]. Until now, treatment effectiveness has primarily been evaluated through clinical outcomes rather than the specific characteristics of the CAR-T cells themselves. Incorporating cell monitoring could offer additional insights by analyzing CAR-T cell expansion and persistence [6,7]. CAR-T cells can be detected using flow cytometry (FC) and/or quantitative PCR (qPCR), the two main methods for evaluating CAR-T in peripheral blood, bone marrow (BM), and cerebrospinal fluid (CSF) [7,8]. Monitoring CAR-T therapy through flow cytometry, digital PCR (dPCR), and the immunophenotypic characterization of circulating CAR-T cells could complement imaging techniques in assessing clinical outcomes [9]. The two main aspects of these cells that determine them to be effective are quantity and quality [10]. A decrease in CAR-T cell dynamics between days 7 and 14 correlates with higher overall relapse rates and lower levels of CAR-T cells present at day +14 post infusion may be associated with early progression [11]. Thus, another analyzed parameter comprises the kinetics of these cells, which may be helpful to guide clinical decisions for patients subjected in the future [12].

Laboratory testing can aid in predicting severity and in CAR-T cell therapy monitoring CRS, ICANS, and other toxicities such as cytopenias/marrow hypoplasia and hypogammaglobulinemia [13]. The measurement of circulating DNA is developmental and promising in predicting cancer relapse after CAR-T cell therapy [13]. The presence of these specific complications underscores the necessity for tailored, extended-term monitoring. Despite many markers, there still exists a critical need to establish universal standards for CAR-T cell analysis, especially in early-phase studies to predict long-term efficacy [14]. Therefore, in this paper, we present the current options and both laboratory as well as clinical parameters and markers to monitor the effectiveness of CAR-T cell therapy in hematological malignancies.

## 2. CAR-T Cell Monitoring 

The primary factor consistently linked to sustained long-term remissions post CAR-T cell therapy is the extent of the initial response to treatment, typically measurable within the initial months following cell infusion [15,16,17,18]. The assessment of disease response following CAR-T cell therapy in patients diagnosed with ALL and NHL typically occurs within the first month of administration [19]. This assessment comprises several components, including peripheral blood (PB) parameters and bone marrow (BM) aspirate examination for morphological changes. It can also bring additional information for the evaluation of Minimal Residual Disease (MRD), which is primarily achieved using imaging techniques like positron emission tomography (PET) scanning accompanied with CT or MR [20,21,22]. In order to obtain standardized MRD evaluation from a PET-CT, the nuclear medicine radiologist has to assign the Deauville score based on both the visual assessment as well as measured SUV max [23]. As there are no official guidelines for imaging-based CAR-T cell therapy follow-up, the use of PET-CT is not reimbursed by some health insurances, which may be a crucial limiting factor [24]. Furthermore, the assessment could be extended to cerebrospinal fluid (CSF) analysis to detect any signs of malignant disease involvement in the central nervous system (CNS) [25]. 

The initial evaluations of disease status in NHL adhere to the International Working Group Response Criteria for Malignant Lymphoma, also known as the Lugano criteria [26]. These assessments are reported as Overall Response Rate (ORR) values and include two primary categories: Complete Response (CR), which incorporates a complete metabolic response even if a residual mass is present, and Partial Response (PR), defined as a reduction of over 50% in the sum of the perpendicular diameters of up to six representative nodes or lesions [26]. Significantly, in cases where NHL patients exhibit PR during the initial disease evaluation, typically conducted around 1–2 months following CAR-T cell therapy, it is noteworthy that more than half of these patients can subsequently experience continued response and transition to a state of CR [27]. Stable disease (SD) and progressive disease (PD) are other disease response categories commonly employed in the setting of NHL [28]. In case of ALL patients, the treatment response evaluation is lacking the standardization of the terminology. Even though it does not have a universal definition in this context, the term CR is also commonly used [29]. According to the consensus guidelines published by the International Myeloma Working Group Immunotherapy Committee in August 2024, the response to CAR-T cell therapy is based on principles similar to response assessment in stem cell transplantation [30]. CR to the multiple myeloma (MM) treatment is defined as negative immunofixation on the serum and urine and the disappearance of any soft tissue plasmacytomas and <5% plasma cells in bone marrow aspirates. Moreover, a stringent complete response is achieved when a patient meets the above-mentioned criteria of CR and also has a κ/λ ratio ≤ 4:1 or ≥1:2 for κ or λ patients, respectively, after counting ≥ 100 plasma cells and there are no clonal cells in bone marrow biopsy detectable by immunohistochemistry [31]. Recently, one of the updates of the criteria introduced the assessment of minimal residual disease (MRD) as a part of the response evaluation process [31]. Following the typical inpatient care regimen designed to address acute toxicities such as CRS, neurotoxicity, and neutropenic fever or infection, the subsequent phase of management primarily revolves around rectifying and providing support for any residual complications associated with CAR-T cell therapy. 

During CAR-T cell therapy, one option is to monitor the levels of CAR-T constructs during treatment. Measuring CAR-T cells’ number and physical parameters associated with their presence can help determine whether a therapy is effective and whether CAR-T cells continue to function appropriately in a given number. An emerging principle of clinical CAR-T cell therapy is the effector-to-target (E:T) ratio [32]. In vitro, this metric pertains to the proportion of CAR-T cells introduced during an experimental setup to tumor cells, where an elevated E:T ratio yields enhanced cytotoxicity [33]. In the context of patient applications, findings from the ZUMA-1 clinical trial, which evaluated CAR-T cell therapy for Diffuse Large B-cell Lymphoma (DLBCL), revealed that the E:T ratio offered a more accurate prognostic indicator of treatment outcomes compared to solely considering tumor burden [34]. The data suggest that the expansion of CAR-T cells demonstrates a positive correlation with increasing tumor burden until it reaches a point of saturation in individuals with the most extensive tumor burden, beyond which expansion diminishes [34,35].

### 2.1. Quantitative Monitoring of CAR-T Cells

#### 2.1.1. Polymerase Chain Reaction

CAR-T cell quantification using polymerase chain reaction (PCR) is based on detecting the DNA copies of the CAR transgene [36]. The PB sample undergoes DNA extraction and then the primers targeted to the specific parts of the modified lymphocyte’s genome coding the CAR are used. One of the common targets is the FMC63 region, which codes the anti-CD19 oligopeptide, responsible for binding to the CD19 protein on tumor cells [11,37]. A crucial advantage of quantitative polymerase chain reaction (qPCR) monitoring is its high sensitivity. According to San Sebastian et al., using qPCR CAR-T cells can be detected in dilutions of up to 1% [11] whereas Wang et al. report the sensitivity of this method to be 0.1% [38]. The qPCR technique is the most reliable method of detection for CAR-T cells in small concentrations such as 0.02–0.01% [39]. To optimize the efficiency of the vector copy number assessment, Schubert et al. have proposed a validated single copy gene (SCG)-based duplex (DP)-qPCR assay (SCG-DP-PCR) [37].

Novel genetic techniques are still being developed to achieve even more accurate monitoring standards. Digital PCR (dPCR) is an advanced version of qPCR that quantifies the absolute copy numbers of the CAR transgene, in contrast to qPCR, which is only an estimation as it is calculated based on the captured fluorescent signal [40,41]. During the two-step dPCR process, the samples are diluted to a concentration of ½ copy per well and each well is analyzed individually for the presence of the PCR product [42]. This method is known for its excellent accuracy and sensitivity, taking into consideration the ability to detect genetically modified cells in small concentrations. Therefore, it is used in hematology for chimerism assessment [7,43,44]. These features are also applicable in the setting of the identification of genetically modified cells [45]. During the monitoring process of B-cell lymphoma patients, dPCR has shown the sensitivity of detecting CAR-T cells in a 0.01% concentration compared to 1% for qPCR, allowing for greater precision in the monitoring process. Cheng et al. report that the digital droplet PCR (ddPCR) is more sensitive than flow cytometry [46]. Digital PCR is a superior technique, taking into consideration that the time-effectiveness and cost-effectiveness of both PCR methods are similar [11]. This highly sensitive method can be especially useful during the first period post infusion, before the expansion of CAR-T cells or during late follow-up, when the CAR-T cell count drops below 0.1% and the use of a high-sensitivity method is recommended [11,40].

As genetic techniques, PCR-based methods only monitor the process of the CAR transgene expression, not the protein synthesis itself. Their main drawback is the risk of the overestimation of the functional CAR-T cell count as they also detect methylated DNA regions, which are not available for transcription; therefore, these cells lack the CAR on their surfaces [47].

#### 2.1.2. Flow Cytometry

Flow cytometry (FC) allows the detection of CAR proteins on the surfaces of particular populations of lymphocytes [36]. Its main advantage is the ability to distinguish CAR-T cells subpopulations [48]. The process begins with an incubation of the patient’s whole PB or BM with a staining product. Then, the stain index is calculated based on mean fluorescent intensity [49]. Sarikonda et al. point out that the selection of appropriate staining regent is crucial for the assay specificity. Reagents comprising fusion proteins like CD19Fc that bind to the antigen-binding site of the CAR construct are unique for each CAR and highly specific. They present low affinity and nonspecific binding. Anti-idiotypic monoclonal antibodies also are highly specific and unique for each CAR, but their main limitation is their high cost. Protein L binds to the kappa light chain of Ig. Although it is a low-cost, universal reagent for scFV-based CAR, it leads to high nonspecific staining. Another low-cost method, which can only be used to monitor CARs with surrogate tags built into the construct design, uses anti-tag monoclonal antibodies [50].

When comparing indirect staining methods like the use of CD19 recombinant protein conjugated with histidine (CD19his) or biotin (CD19bio) to the direct method using CD19 conjugated with the fluorochrome (CD19-FITC), they appear to be more precise monitoring tools. In studies, the unspecific binding for CD19his and CD19bio occurred at 0.06% and 0.07%, respectively, while for CD19-FITC, it reached 0.53%. Overall, all of the above-mentioned methods can detect CAR cells with good discrimination between the CD19-positive and -negative cell populations. As the use of CD19his is more cost-effective than that of CD19bio, it is thought to be the best staining method during CAR-T cell therapy monitoring [49]. A comparison of three FC detection methods for BCMA-CAR-T cells proved that all of them are capable of clearly distinguishing between BCMA-CAR-positive and -negative T cells. Moreover, the sensitivity also did not differ significantly. The BCMA detection reagent had a very low false-positive staining of 0.04 ± 0.02% compared to a PE-labeled human BCMA peptide, which showed a false-positive staining of 0.25 ± 0.06%. Overall, the polyclonal anti-human IgG PE antibody showed the highest false-positive staining of 7.2 ± 9.2% as it is not a BCMA-specific reagent and it binds to the CH2-CH3-hinge region of the CAR [39]. Schanda et al. also compared the sensitivity and specificity of nonspecific (Protein L and F(ab’) fragment) detection reagents to those specifically binding to the CD19 binding site of the scFv. They showed that the CD19 CAR detection reagent yielded the highest frequencies of CAR-T cells. Moreover, it showed almost no unspecific binding, which should be especially important for cases with low CAR-T cell concentrations. Contrary to the above-mentioned advantages of specific staining, the main limitation of this method is its high cost [7].

Generally, the sensitivity of FC ranges from 0.1% to 0.01% [11]. To perform with the highest accuracy, the FC laboratory should meet the regulatory requirements and the analysis should be performed by fully trained staff [50]. Classical FC sample processing takes around 30 min for cell lysis with a total turnaround time of 1 hour, a 20 min hands-on time, and a relative final price per patient of 20 EUR. Time-effectiveness is a crucial advantage over dPCR, which comes with a total turnaround time of 4h and hands-on time of 30 min with a similar cost per patient [11]. To achieve higher sensitivity, the use of time-consuming bulk lysis proceedings is necessary [11]. An alternative method called image cytometry is performed on 96-well plates containing patients’ undisrupted cells. This modification to the standard flow cytometry method can lead to higher time efficiency and quality in the results. Moreover, it also allows for the analysis of the samples over time [38].

Most researchers agree that flow cytometry and qPCR/dPCR are complementary methods [7,39,46]. The use of qPCR or even dPCR is recommended in cases of small concentrations of CAR-T cells to achieve more accurate results about CAR-T cell persistence [8,39].

### 2.2. Monitoring of the Activity of CAR-T Cells

The monitoring process of CAR-T cell therapy should also cover the activity of the infused cells. In patients undergoing CAR-T cell therapy, we aim not only for the modified lymphocytes to be present in their PB but also to preserve their cytotoxic function against tumor cells. It is proposed that functional persistence is measurable by assessing B cell aplasia, which is associated with a lower risk of relapse [36]. There are a wide variety of assays used for measuring CAR-T-mediated cytotoxicity. Four of the most commonly used include the chromium (51Cr) release assay (Cr assay), the luciferase-mediated bioluminescence imaging (BLI) assay, the impedance-based assay, and the flow cytometry assay (FCA) [32].

Dating back to 1968 is what used to be the gold-standard method—the Cr assay. The release of radioactive chromium isotopes to the medium took place during the loss of the integrity of the cell membrane in pre-labelled target cells, which were killed by the effector cells. The radioactivity of the supernatant, which correlates with the number of killed cells, was usually measured after a few hours of incubation. What is worth highlighting is that the Cr assay involved the use of a radioactive reagent, which put the staff’s health at the risk of radiation [51]. Therefore, it has been replaced in recent years with modern, more complex methods.

During the luciferase-mediated BLI, the target cells are transduced with the luciferase reporter gene, leading to bioluminescence, which acts as a marker of cell viability. Decreased bioluminescence, detected and measured by a luminometer, is a proof of effector-cell cytotoxic activity resulting in target cells’ death [52,53]. With the application of biotechnology to transduce cells, the BLI assay is not only easier to perform than the Cr assay but also more time-efficient. Additionally, the bioluminescence can be measured at many time points, and it is characterized by a higher signal-to-noise ratio, leading to more precise results. Taking all of the above-mentioned advantages into consideration, BLI turns out to be a safe, radiation-free alternative to the gold-standard method [52].

A fully automated, kinetic-based method for measuring cytotoxicity is the impedance-based assay. The procedure involves seeding the target cells on microtiter plates integrated with microelectrodes at the bottom of the well [54]. The real-time cell electronic sensing (RT-CES) system is responsible for detecting the electrical impedance, which depends on the number, morphologic aspect viability, and degree of adhesion of the cells [55,56]. When comes to effector-mediated target cell death, it is followed by structural changes in the cell’s cytoskeleton, leading to the loss of adhesion to the plate depicted by a decrease in measured impedance [54]. Cytotoxicity assessment using RT-CES was found to be equally sensitive when compared to the use of a neutral red uptake assay at specific time points [55]. The results obtained from this system also correlate with those from MTT assays and crystal violets [54]. Both Erskine et al. as well as Peper et al. confirmed that the impedance-based assay’s sensitivity is higher compared to that of the Cr assay [57,58]. Moreover, this is a radiation-free method allowing for real-time measurements [32]. It can be particularly useful for CAR-T cell therapy monitoring as Eugene-Norbert et al. proposed an optimized cytotoxicity assay with enhanced specificity towards CD19 to overcome the problem of alloreactivity. This improved version of the impedance-based assay measures only cytotoxicity against tumor cells expressing CD19 on their surface and its sensitivity is comparable to FC and microscopy [59]. The highly sensitive impedance-based xCELLigence assay is the most commonly used to evaluate the cytotoxic activity of CAR-T cells [60,61].

As mentioned previously, FC can function as a tool for CAR-T cell persistence monitoring. However, it can not only measure the number of cells but also detect their phenotypes and divide them into subpopulations [8,36]. Therefore, it can separate the effector and the target cells based on the differences in their sizes and granularities. FC allows for the use of a wide variety of monoclonal antibodies, which target specific proteins of the cell. Different approaches to cell death detection using FC have been proposed by researchers. Riccardi et al. showed that it can be evaluated using DNA-intercalating fluorescent agents like propidium iodide or 7-aminoactinomycin D [62]. Liu et al. developed an assay based on a fluorescent substrate for the caspases [63], similar to Packard and Komoriya, who focused on the activation of intracellular proteases [64].

Along with the rapid development of cellular therapies, there is an increasing need for the development of precise and optimal monitoring tools. One of the innovative techniques is carboxyfluorescein diacetate succinimidyl ester staining combined with FC assay. The use of this particular reagent allows for following the number of cell divisions each cell undergoes. Therefore, when combined with the ability of FC to divide the cells into subpopulations, it gives an important insight into the target cell population [65]. FC is a powerful tool to perform multiparameter analysis, which can be used for the detailed monitoring of CAR-T cell therapy. An antibody panel called CAR-T3 for assessing the effector function of anti-CD19 CAR-T cells has been proposed by Blache et al. It is a 13-colour/15-parameter assay targeting both intra- and extracellular proteins. The authors suggest that in the future, this strategy might be combined with growing machine learning technologies to develop even more complex quality control tools for CAR-T cell products [48].

In summary, the main advantage of FC over other assays is its ability to measure the cytotoxicity on heterogeneous targets [32]. Moreover, the FC assay is more sensitive than the outdated Cr assay [63] and allows for fast analysis at a single-cell level [48].

### 2.3. Phenotyping of T-Cell Subsets 

One of the most essential methods involves analyzing the phenotypic composition of CAR-T cells from blood samples by FC [66,67]. Various studies indicate a connection between the attributes of T cells in the infused product and the ensuing CAR responses [68,69,70,71]. The essential information is comparing the proportion between CD4+ and CD8+ CAR-T cells. When contrasting the utilization of separate T-cell subsets for CAR-T cells, the concurrent application of CD4+ and CD8+ subsets demonstrates synergistic anti-cancer properties [72]. A recent study tracking the progress of two patients a decade after CAR-T cell therapy found that over 99% of the CAR-T cells were CD4+ while fewer than 1% were CD8+. These findings suggest a significantly increased presence of CD4+ T cells in the body over time [67,73,74]. The results show that CAR-T cell expansion, particularly of the CD4+ subtype, is associated with a better response and higher toxicity [75].

Another important step is analyzing the CAR-T subsets, which can vary depending on the individual and culture methods used [66,67,76]. The proliferative and survival capacities abilities of T cells are influenced by their stage of differentiation. There is a significant link between T cell proliferation, survival, and anti-tumor effectiveness once these cells enter into the body [77,78]. The initial immunophenotype of the cells used to generate CAR-T cells is associated with the treatment outcomes. For instance, sustained remission is often linked to the presence of CD27+/CD45RO−/CD8+ T cells with memory-like characteristics [71,79]. The phenotypic analysis of circulating CAR-T cells requires a sufficient number of CAR-T cells to identify rare subsets [80,81].

Consequently, during the persistence phase of CAR-T cell therapy, many patient samples are not evaluable as they are close to the detection limit [3,81]. The optimal time for this analysis is within 14 days after CAR-T cell administration, during which there is a vigorous expansion phase followed by a rapid contraction phase. After this, the persistence phase begins, characterized by a gradual decline in CAR-T cell counts [82]. 

T-cell subsets are categorized based on their differentiation levels and can be differentiated by the presence of various surface markers. The established theory of T-cell differentiation suggests that when quiescent naive T cells (T_N_) undergo differentiation, they transform into effector T cells (T_EFF_), which are specialized killer cells responsible for cytotoxic effects [79,83]. The T_EFF_ phenotype was initially considered optimal for T-cell therapies because of their strong killing capabilities. Nonetheless, T_EFF_ cells struggle to increase and endure in a living organism [71,84]. Thus, the CAR-T cell therapy field is shifting its focus toward developing protocols that maintain T cells in a less differentiated state. Initially, the efforts were concentrated on creating T cell products with two distinct cell phenotypes: naive central memory T cells (T_CM_) and effector memory T cells (T_EM_). T_CM_ cells are known to have improved replicative capacity but limited effector functions. The second type, T_EM_ cells, are more cytolytic, express chemokine receptors, and possess adhesion molecules necessary for migration to peripheral tissues but have the worst replication capability [85]. On the contrary, in vitro studies have shown that T_CM_ cells express fewer genes linked to effector functions than T_EM_ cells [86]. Experimental models before clinical trials have been employed to assess CAR-T cells’ endurance and operational attributes originating from memory and naive T-cell groups. Findings have indicated that CAR-T cells crafted from CD4+ and CD8+ T_N_ and T_CM_ subsets exhibit heightened tumor-fighting potency and proliferation compared to those originating from T_EM_ [72]. Additionally, it was found in a study that T_SCM_ CAR T cells were observed to provide enduring anti-leukemic reactions in xenograft models [87] and in humans [88]. The optimal T cell composition remains uncertain; however, fewer numbers of T_SCM_ or T_CM_ cells appear crucial for a response to adoptive cell therapies [72,77,86]. Thus, the potential monitoring of the composition of the infused product through these therapies could yield an additional predictor factor of effectiveness and remission. So far, guidelines have been developed for creating CAR-T cell treatments with abundant T cells resembling memory cells. Consequently, a phase I trial has showcased the safety and viability of using these T_CM_-like CAR-T cells [89].

The cellular composition of T-cell subsets can influence CAR-T cell function and the currently available commercial CAR-T are different. Improved outcomes with CAR-T cell therapy have been seen in patients in whom the CAR-T product contains a greater proportion of less differentiated T-cell subsets [71,89,90]. Additionally, the effectiveness of T cells treatment hinges on their ability to proliferate and maintain prolonged functionality [80]. Clinical findings have indicated that the enduring in vivo presence of adoptively transferred CAR-T cells necessitates the presence of less specialized memory T cells. Conversely, positive treatment responses are associated with T_N_, T_CM_, and stem-like memory (T_SCM_) lymphocytes, attributed to their capacity for proliferation and extended longevity [22]. Effector T-lymphocyte (T_E_) subsets have a limited self-renewal capacity, a reduced ability to home to tumor sites, and lower survival rates compared to memory lymphocyte T (T_M_) subpopulations [91,92,93]. Preclinical studies have suggested that CAR-T cells derived from T_N_ and T_CM_ cells demonstrate greater anti-tumor activity and proliferation then those originating from effector memory T lymphocytes [72]. These findings highlight the importance of naive and memory T cells in CAR-T therapy due to their ability to sustain prolonged proliferation and persistence in vivo [94,95]. The surface markers linked to different T-cell differentiation stages are presented in Table 1 [22,72,96,97,98,99,100,101,102,103]. The studies above have indicated that the critical feature of influential lymphocyte groups is a high potential for differentiation and proliferation. 

Regulatory T cells (Tregs) express CD95+ and CD127low and are observed in the TME in inflamed and non-inflamed tumors [104]. Increased numbers of CD4(+) Tregs expressing the transcription factor FoxP3 in malignant tumors promote tumor progression by suppressing effective anti-tumor immunity. It has been shown that decreased ratios of CD8(+) T cells to Tregs among tumor-infiltrating lymphocytes are correlated with poor prognosis in various types of human cancers [105]. The phenotypic and functional diversity of intratumoral immunosuppressive regulatory T cells (Tregs) can impact their response to therapy and may offer new targets to modulate specific Treg subsets [106]. Therefore, the final CAR-T cell product should be defined by phenotyping T cells to improve their anti-tumor efficacy in vivo [67,104,105,106].

## 3. Biomarkers and Parameters for Monitoring CAR-T Cell Therapy 

Biomarkers constitute a crucial component of precision medicine, enabling the objective characterization of biological processes [107]. Beyond simply enhancing the understanding of a disease, they can act as predictive, prognostic, or therapeutic markers. Different types of biomarkers offer distinct insights into the disease process [108]. In the context of CAR-T cell therapy, they are particularly valuable for evaluating the treatment’s effectiveness [109]. One of the earliest biomarkers this therapy uses is lactate dehydrogenase (LDH), which is consistently linked to a more significant tumor burden. This enzyme belongs to the acute-phase protein (APP) group, which includes C-reactive protein (CRP) or ferritin. Thus, each is associated with developing CRS and ICANS [110,111]. It has been shown that higher LDH levels are associated with worse outcomes in patients with B-cell malignancies undergoing CAR-T cell therapy [112,113,114]. Elevated levels of CRP, ferritin, and D-dimer have been shown to correlate with more severe CRS while lower levels have been associated with a better response to tocilizumab and corticosteroids [115].

### 3.1. Immune Checkpoint Molecules

**Figure 1:**
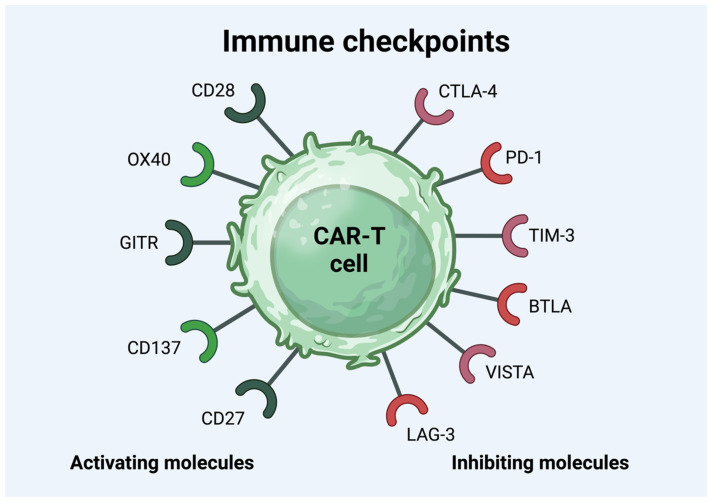
The immune checkpoint molecules on the surface of CAR-T cell. Abbreviations: Glucocorticoid-induced tumor-necrosis-factor-receptor-related protein (GITR), cytotoxic T-lymphocyte-associated antigen 4 (CTLA-4), programmed death-1 (PD-1), T-cell immunoglobulin and mucin domain 3 (TIM-3), B and T lymphocyte attenuator (BTLA), V-domain immunoglobulin suppressor of T cell activation (VISTA), lymphocyte activation gene 3 (LAG-3), and chimeric antigen receptor T (CAR-T) [122].

Immune checkpoint inhibitory molecules, such as PD-1, LAG-3, and TIM-3, are significant indicators for forecasting the effectiveness and longevity of CAR-T cell therapy [116]. These markers are most extensively studied and are associated with T cell exhaustion, leading to an inadequate response to CAR-T cell treatment [117,118]. PD-1, a biomarker found on activated T cells, NK natural killer cells, and B cells, can suppress T cell proliferation, cytokine secretion, and cytotoxic activity, leading to the escape of tumor cells from the immune system [119,120,121,122]. It was shown in a study that functional and dysfunctional responders had similar frequencies of PD-1+ CD4+ CAR-T cells and PD-1+ CD8+ CAR-T cells. In contrast, the dysfunctional response group had a significantly higher percentage of LAG-3+ T cells [123]. LAG-3 and TIM-3 represent two emerging immune checkpoint proteins found on various immune cell varieties, and they share a standard function in lowering T cell activity [124,125]. It was shown in a study that the high expression of LAG-3 was associated with early therapeutic failure [123]. The main immune checkpoint molecules are presented below in Figure 1. 

### 3.2. Cytokines

Cytokines have prompted improvements in CAR-T cell therapy, boosting their proliferation, reversing T cell exhaustion, and enhancing their anti-tumor capabilities [69,114,126,127,128,129]. Various inflammatory cytokines, including IL-6, IL-7, IL-8, IL-12, IL-15, IL-18, IFN-γ, and TNF-α, have demonstrated the ability to amplify T-cell cytotoxic functions [69,114,126,127,128,129]. Moreover, monitoring the plasma concentrations of these molecules is of major clinical significance as they are biomarkers of CRS, a life-threatening complication described profoundly in a separate paragraph below [130,131]. It has also been proposed that such elevated concentrations may depict the possibly happening tumor lysis [132]. Conversely, IL-10, TGF-β, and IL-4 are suppressive cytokines that may impair CAR-T cells [133,134]. Moreover, the release of various cytokines and chemokines by versatile T cells, such as IFN-γ, MIP-1, IL-8, granzyme B, IL-17A, and IL-5, can alleviate the immunosuppressive effects induced by the tumor microenvironment (TME) and enhance the therapeutic outcomes in CD19 CAR-T cell treatment [69]. This release has the potential to improve CAR-T cell anti-tumor effectiveness by fostering the growth of CD8+ T cells while decreasing the presence of immunosuppressive cells [128,135].

It has been proposed by Klaver et al. that during the monitoring process of CAR-T cell therapy, the plasma levels of IFN-γ and IL-6 should be measured as these two cytokines serve as indicators for T-cell persistence. The authors have also highlighted the need for further studies on this topic to determine whether the levels of these cytokines correlate with anti-tumor activity [136]. 

### 3.3. ctDNA

Our recent understanding acknowledges that the blood of cancer patients contains circulating tumor DNA (ctDNA), which can offer insights into tumor characteristics and treatment effectiveness. CtDNA exists as fragmented pieces, with a predominant size peak at 166-167 base pairs [137,138]. CtDNA can be a valuable tool for tracking the response to CAR-T cell therapy, with day-28 ctDNA levels proving more effective than PET imaging in predicting future relapses [107]. In a different research investigation, the assessment of ctDNA on day 7 post infusion successfully differentiated between different early therapy outcomes. At the 3-month follow-up, the majority of patients who had achieved a >5-fold molecular response went into CR in opposite to the <5-fold molecular response group, where CR was not reported. These promising results highlight the need for future studies validating the association between the early therapy outcomes and long-term effectiveness of CAR-T cell therapy [70]. In an ideal scenario, the early assessment of the tumor response following CAR-T cell treatment would enable timely intervention in cases of inadequate tumor elimination. For instance, if day 7 ctDNA levels correlated with poor outcomes, these patients could be considered for additional CAR-T cell dosing [70].

## 4. Monitoring of the Adverse Effects of CAR-T Cell Therapy—Current Practice and Future Options

### 4.1. Cytokine Release Syndrome

CAR-T cell immunotherapy releases large numbers of cytokines, leading to cytokine release syndrome (CRS) and neurotoxicity [139,140,141,142,143,144,145]. CRS is a clinical syndrome resulting from widespread immune activation, associated with the expansion of CAR-T cells and significant increases in serum inflammatory markers and cytokines [131]. Initial clinical manifestations of CRS include tachycardia, hypotension, hypoxia, nausea, and vomiting, and it can progress to life-threatening complications such as severe hypoxia or organ dysfunction [146,147]. The incidence of CRS in patients treated with CAR-T cells ranges from 37% to 93% [27,28,148,149]. The onset of CRS varies depending on the CAR-T cell product and patient population, typically peaking 2–7 days after infusion, though delays of up to 3 weeks have been reported [146,150]. Consequently, mortality is observed in up to 9.1% of cases [146,151].

The development of CRS can lead to various clinical symptoms and elevated concentration of biomarkers such as IL-1β, IL-2, IL-6, IL-7, IL-8, IL-10, IL-15, TNF-α, and IFN-γ [130]. While some of them are only utilized in research settings, parameters like lactate dehydrogenase, uric acid, ferritin, and CRP are used on a regular basis in the monitoring process [152]. The patient’s symptoms used for the classification of the grades of CRS are presented in Table 2, derived from the American Society for Transplantation and Cellular Therapy (ASTCT) consensus guidelines for CRS grading [139,153,154,155]. In the case of CRS, most centers require reevaluation every 4 hours for grades 1 and 2, and more frequent assessments, ranging from 1 to 2 hours for grades 3 and 4 CRS, following established guidelines [156,157]. 

CRS is primarily treated using IL-6 inhibitors such as tocilizumab for milder cases and corticosteroids for severe, persistent, or more severe cases of CRS [131,157,158,159,160,161,162]. While there are limited data on other treatments, alternative IL-6 blockers like siltuximab and clazakizumab may be considered for cases where tocilizumab is ineffective. However, there have been no direct comparative studies assessing the effectiveness of these IL-6 blockers. Anakinra, an IL-1 receptor antagonist, has been shown to alleviate CRS in some recipients of CAR-T-cell therapy experiencing severe CRS [131,157,158,159,160,161,162,163].

### 4.2. Immune-Effector-Cell-Associated Neurotoxicity Syndrome

**Figure 2:**
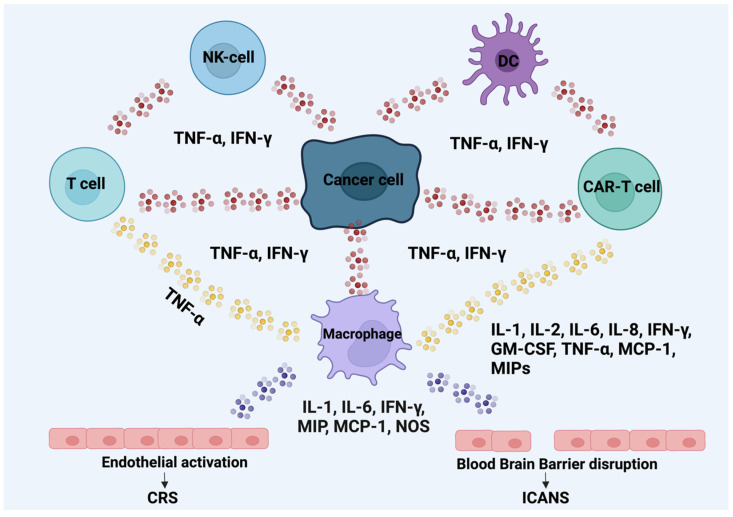
Mechanism leading to CRS and ICANS. The red dots indicate TNF-α and IFN-γ, yellow dots indicate TNF-α, and purple dots indicate IL-1, IL-6, IFN-γ, MIP, MCP-1, and NOS. The interactions between them are bidirectional. Abbreviations: tumor necrosis factor α (TNF-α), interferon gamma (IFN-γ), macrophage inflammatory proteins (MIP), monocyte chemotactic protein 1 (MCP-1), nitric oxide synthases (NOS), chimeric antigen receptor (CAR), cytokine release syndrome (CRS), immune-effector-cell-associated neurotoxicity syndrome (ICANS), and blood–brain barrier (BBB) [143,144,145].

ICANS is described as a condition involving the central nervous system (CNS) that is triggered by immune effector therapies that activate or involve both natural and infused T cells along with other immune effector cells. Figure 2 presents the main mechanism leading to the CRS and ICANS. Symptoms or signs can advance and may involve aphasia, changes in consciousness, declines in cognitive abilities, muscle weakness, seizures, cerebral edema, headaches, compromised attention and consciousness, lethargy, agitation, hallucinations, tremors, aphasia, encephalopathy, and seizures [160,163]. The median time to onset is 4 days after infusion [150]. A comprehensive evaluation involves clinical manifestations and a lumbar puncture for cerebrospinal fluid (CSF) analysis, neuroimaging, and EEG to assess the severity of ICANS-related damage and to exclude alternative organic factors [157,160]. Indeed, the expeditiousness of CAR-T cells’ in vivo expansion has been correlated with the initiation and intensity of ICANS [164,165,166,167]. In some cases, ICANS symptoms may coincide with CRS, particularly when toxicities reach more severe grades [168,169]. 

The diagnosis and severity of ICANS rely on clinical manifestations and lumbar puncture, neuroimaging, and EEG [157]. So far, numerous risk factors associated with CAR-T cell neurotoxicity have been delineated, including pre-treatment disease burden, the in vivo expansion of CAR-T cells, the onset of early and severe CRS, and the administered dose of CAR-T cells [2]. 

In the clinical assessment of ICANS, laboratory analysis encompasses biomarkers analogous to those employed for CRS. Numerous clinical studies have associated various cytokines with the onset and intensity of ICANS. In studies, patients with B-cell lymphoblastic leukemia following CAR-T cell therapy presented elevated levels of IL-1α, IL-2, IL-3, IL-5, IL-6, IL-8 IL-10, IL-15, IFN- γ, procalcitonin, CRP, G-CSF, GM-CSF, and MCP-1, and their levels were linked to the severity of neurotoxicity [159,170]. In particular, a close association persists between the emergence of ICANS and elevated IL-6 levels following treatment [22,28,171]. Alongside high levels of IL-6 in patients experiencing ICANS, elevated levels of IL-15, a cytokine known for promoting the proliferation and activation of T and NK cells have been presented [166,170,171,172,173,174,175,176]. Low platelet counts before treatment have also been linked to an increased risk of CRS [170]. In fact, low platelet levels might serve as biomarkers for blood–brain barrier disruption, which has been previously connected to the development of CAR-T-related ICANS [166]. The myeloid proliferation and activation of the cytokine GM-CSF represent a frequently observed blood marker [16,170,174,176]. The function of GM-CSF in bolstering the activity of inflammatory macrophages and monocytes leads to the production of CRS and ICANS [177]. Santomasso et al. identified initial elevations in IL-6, IL-10, GM-CSF, and G-CSF levels, excluding ferritin, in individuals undergoing CAR-T cell therapy who subsequently manifested ICANS. In contrast, Faramand et al. noted baseline elevations in IL-6 and ferritin among patients treated with CAR-T cells who later experienced ICANS. Overall, baseline IL-6 and ferritin elevations are indicators of proinflammatory state and are possible ICANS risk factors [178,179]. Additionally, one study created a successful forecast model to predict the risk of ICANS following CAR-T cell therapy. This study included only a few parameters: maximum daily temperature, CRP, IL-6, and procalcitonin. It presented that even a few relatively simple markers could be beneficial for monitoring patients treated with CAR-T cell therapy, predicting the risk of ICANS [180]. 

Two scales have been created to assess the severity of ICANS: the CAR-T-cell-therapy-associated TOXicity 10 (CARTOX-10) and the immune-effector-cell-associated encephalopathy (ICE) scales [149,157,181]. The ASTCT has issued guidelines for the consensus grading of ICANS, employing the ICE score, alongside considerations such as a diminished level of consciousness, seizures, motor manifestations, and cerebral edema [153,182]. 

Currently, according to the 2021 best-practice recommendations of the European Society for Blood and Marrow Transplantation (EBMT) and the Joint Accreditation Committee of ISCT and EBMT (JACIE) and the European Haematology Association (EHA), anti-seizure prophylaxis should only be used in high-risk cases, rather than in every patient after CAR-T cell infusion. The mainstay of treatment ICANS comprises supportive care and corticosteroids. Additionally, levetiracetam and benzodiazepines should be administered to patients presenting with seizures in clinical examination or detectable by EEG [2]. Several additional treatment options are currently being studied in clinical trials, including anakinra, lenzilumab, and defibrotide [183]. 

### 4.3. Hemophagocytic Lymphohistiocytosis 

Hemophagocytic lymphohistiocytosis (HLH) is marked by the accumulation of histiocytes and lymphocytes in organs such as the skin, spleen, and liver, leading to the destruction of other blood cells. It is relatively uncommon, with an incidence rate of about 3.5% [184]. CAR-T-induced HLH can present with symptoms like fever; the enlargement of the spleen and liver; swollen lymph nodes; skin rashes; jaundice; respiratory problems such as coughing and difficulty breathing; gastrointestinal issues like abdominal pain, vomiting, and diarrhea; and neurological symptoms including headaches, difficulty walking, visual problems, and weakness. The diagnostic criteria for CAR-T-cell-related HLH suggest ferritin levels above 10,000 ng/mL, accompanied by at least two organ dysfunctions, which may include hemophagocytosis in the bone marrow or organs or severe transaminitis, kidney impairment, or grade 3 or higher pulmonary edema. Corticosteroids and IL-6 inhibitors have been employed to manage HLH in CAR-T-cell therapy patients experiencing an organ toxicity of grade 3 or higher. Etoposide is another potential treatment option, though there are limited data available specifically for CAR-T-cell therapy recipients [185]. Anakinra has been administered to CAR-T-cell therapy recipients with refractory HLH, but its clinical effectiveness remains uncertain [163].

### 4.4. Cytopenias/Marrow Hypoplasia

Cytopenias are prevalent after CAR-T cell therapy, with neutropenia emerging as the most frequently observed variety [186,187]. Cytopenias, such as anemia, thrombocytopenia, leukopenia, and neutropenia, are characterized by a decrease in the number of mature blood cells. Symptoms associated with CAR-T-cell-therapy-induced cytopenias can include fatigue, weakness, shortness of breath, difficulty concentrating, dizziness or lightheadedness, cold extremities, frequent infections, fever, and bleeding [188]. Their incidence ranges between 20 and 80% among patients and may extend beyond 30 days post administration [2,189]. In a systematic analysis of post-CD19 CAR-T cell therapy, the frequencies of anemia, thrombocytopenia, and neutropenia of all grades were reported as 65%, 55%, and 78%, respectively. Age, gender, disease, prior lines of therapy, and the target and costimulatory domain have been identified as influential determinants in cytopenias after CAR-T cell therapy [190]. In the majority of cases, cytopenias tend to resolve spontaneously with time. In persistent or delayed cytopenias, conducting a bone marrow biopsy is advisable to assess the potential presence of secondary bone marrow malignancies [190]. The management of cytopenias encompasses the administration of packed red blood cell (PRBC) and platelet transfusions, as well as the utilization of growth factors like eltrombopag to address persistent severe thrombocytopenia. In cases of severe neutropenia, granulocyte-colony-stimulating factor (G-CSF) may also be employed to stimulate the production of neutrophils [190,191,192,193]. 

### 4.5. B-Cell Aplasia and Hypogammaglobulinemia

An indirect measure of anti-CD19 CAR T-cell presence is B-cell aplasia (BCA), defined as a disorder caused by the depletion or absence of B cells. Related symptoms of CAR-T-induced BCA include low B-cell counts and low immunoglobulin levels [188,194]. BCA and hypogammaglobulinemia are expected on-target, off-tumor effects of CD19+ targeted CAR-T cells. The disorder is caused by malignant B cells expressing CD19 [195]. New CAR-T cell constructs targeting the BCMA protein used for MM treatment deplete both malignant cells and normal B cells, which reach late stages of differentiation including as plasma cells producing immunoglobulins [196]. A comparison of the clinical trials submitted to the FDA during the products’ registration showed that the anti-BCMA CAR-T cell construct resulted in the highest rate of hypogammaglobulinemia (41%) [195]. Hypogammaglobulinemia, defined as IgG < 400 mg/dL, is more frequent in children compared to adults and may occur for up to four years [195,197,198]. Hypogammaglobulinemia resulting from BCA can be associated with an increased risk of infections [199,200]. Significantly, cytopenias and hypogammaglobulinemia are associated with significant morbidity and mortality after CAR-T cell therapy [201,202,203]. The management comprises immunoglobulin replacement therapy, which should not be used as a prophylaxis but is recommended in patients suffering from recurrent or severe bacterial infections. Children and patients treated with anti-BCMA CAR-T cell products may require more intensive IgG replacement [202,204]. It is recommended to conduct a baseline evaluation of lymphocyte subsets and immunoglobulin levels in all adult patients prior to lymphodepletion chemotherapy, followed by monthly monitoring thereafter [149,205]

## 5. Conclusions and Future Direction

Despite its challenges, CAR-T cell therapy has brought new hope to patients with hematological malignancies. So far, the focus has primarily been on monitoring the clinical effects of CAR-T cell treatment. Numerous parameters and biomarkers hold promise for the early, reliable, and rapid identification of patients most at risk for CRS or ICANS. The therapeutic spectrum of cancer immunotherapy may be expanded by identifying new therapeutic targets. Also, the effectiveness of immune precision therapy depends on conditions in the TME. Advanced imaging technologies are crucial for monitoring the distribution and persistence of CAR-T cells. Therefore, the detailed analysis of immune cell functions combined with a better understanding of the generation of the T-cell subsets may be important in effective monitoring CAR-T cell therapy. 

## Figures and Tables

**Table 1:** Surface markers associated with T-cell differentiation stages. Abbreviations: naive T cells (T_N_), stem-cell-memory T cells (T_SCM_), central memory T cells (T_CM_), effector memory T cells (T_EM_), and effector T cells (T_EFF_).

T-Cell Subset	Surface Marker Expression	References	
Naive T Cells (TN)	CD45RO-, CCR7+, CD45RA+, CD62L+, CD95-,	[22,72,96,97,98,99]	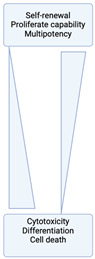
Stem-cell-memory T cells (TSCM)	CD45RO+, CCR7+, CD45RA+, CD62L+, CD95+	[22,72,96,97,99]	
Central memory T cells (TCM)	CD45RO+, CD45RA-, CCR7+, CD62L+, CD95+	[22,72,96,97,99,100]	
Effector memory T cells (TEM)	CD45RO+, CD45RA-, CCR7-, CD62L-, CD95+	[22,72,96,97,99,100]	
Effector T cells (TEFF)	CD45RO-, CD45RA+, CCR7-, CD62L-, CD95+	[72,97,103]	

**Table 2:** American Society for Transplantation and Cellular Therapy (ASTCT) CRS consensus grading.

CRS Parameter	Grade 1	Grade 2	Grade 3	Grade 4
Fever	Temperature ≥ 38 °C	Temperature ≥ 38 °C	Temperature ≥ 38 °C	Temperature ≥ 38 °C
Hypotension	None	Not requiring vasopressors	Requiring a vasopressor with or without vasopressin	Requiring multiple vasopressors (excluding vasopressin)
Hypoxia	None	Requiring low-flow nasal cannula or blow-by	Requiring high-flow nasal cannula, facemask, nonrebreather mask, or Venturi mask	Requiring positive pressure (e.g., CPAP, BiPAP, intubation and mechanical ventilation)

Fever is defined as a temperature ≥38 °C not attributable to any other cause. In patients who have CRS and then receive antipyretic or anticytokine therapies such as tocilizumab or steroids, fever is no longer required to grade subsequent CRS severity. In this case, CRS grading is driven by hypotension and/or hypoxia. (Note: CRS—cytokine release syndrome, CPAP—continuous positive airway pressure, and BiPAP—bi-level positive airway pressure.)
